# Supplementary material for: Regional specialization of movement encoding across the primate sensorimotor cortex
Source: Nat Commun. 2025 Jul 1;16:5729. doi: 10.1038/s41467-025-61172-8 (PMC12216890; doi:10.1038/s41467-025-61172-8)
Supplement: Supplementary file 1 — Supplementary Information [file 41467_2025_61172_MOESM1_ESM.pdf]

Supplementary information to:

Regional specialization of movement encoding across the primate sensorimotor cortex

Simon Borgognon[1,2,3,4,5,&], Nicolò Macellari[1,2,3,6,&], Alexandra M. Hickey[1,2,3,4], Matthew G. Perich[7,8], Houman Javaheri[9], Rafael Ornelas-Kobayashi[1,2,3], Maude Delacombaz[1,2,3,4], Christopher Hitz[1,2,3], Florian Fallegger[10], Stéphanie P. Lacour[10], Erwan Bezard[11], Eric M. Rouiller[4], Jocelyne Bloch[1,2,3], Tomislav Milekovic[1,2,3,4,15,*], Ismael Seáñez[1,2,3,11,12,13,15,*], Grégoire Courtine[1,2,3,15,*]

1 NeuroX Institute and Brain Mind Institute, School of Life Sciences, Swiss Federal Institute of Technology (EPFL), Lausanne, Switzerland

2 Department of Clinical Neuroscience, Lausanne University Hospital (CHUV) and University of Lausanne (UNIL), Lausanne, Switzerland

3 .NeuroRestore, EPFL/CHUV/UNIL, Lausanne, Switzerland

4 Department of Neurosciences and Movement Sciences, University of Fribourg, Fribourg, Switzerland

5 Center for the Neural Basis of Cognition, Department of Bioengineering, University of Pittsburgh (PA), USA

6 Rehab Neural Engineering Labs, Department of Physical Medicine and Rehabilitation, University of Pittsburgh, Pittsburgh (PA), USA

7 Department of Fundamental Neuroscience, University of Geneva, Geneva Switzerland

8 Department of Neuroscience, Icahn School of Medicine at Mount Sinai, New York, USA

9 Institute of Neuroinformatics, ETH Zürich and University of Zürich, Switzerland

10 Bertarelli Foundation Chair in Neuroprosthetic Technology, Institute of Microengineering, Institute of Bioengineering, Center for Neuroprosthetics, École Polytechnique Fédérale de Lausanne (EPFL), Lausanne, Switzerland

11 University of Bordeaux, CNRS, IMN, UMR 5293, F-33000, Bordeaux, France.

12 Department of Biomedical Engineering, Washington University in St. Louis, St. Louis, USA

13 Department of Neurosurgery, Washington University School of Medicine, St. Louis, USA

14 Division of Neurotechnology, Washington University School of Medicine, St. Louis, USA

15 Jointly supervised this work

&, Equal contribution
* Corresponding authors

Corresponding authors:
Grégoire Courtine, PhD
gregoire.courtine@epfl.ch

Ismael Seáñez, PhD
ismaelseanez@wustl.edu

Tomislav Milekovic, PhD
tomislav.milekovic@epfl.ch

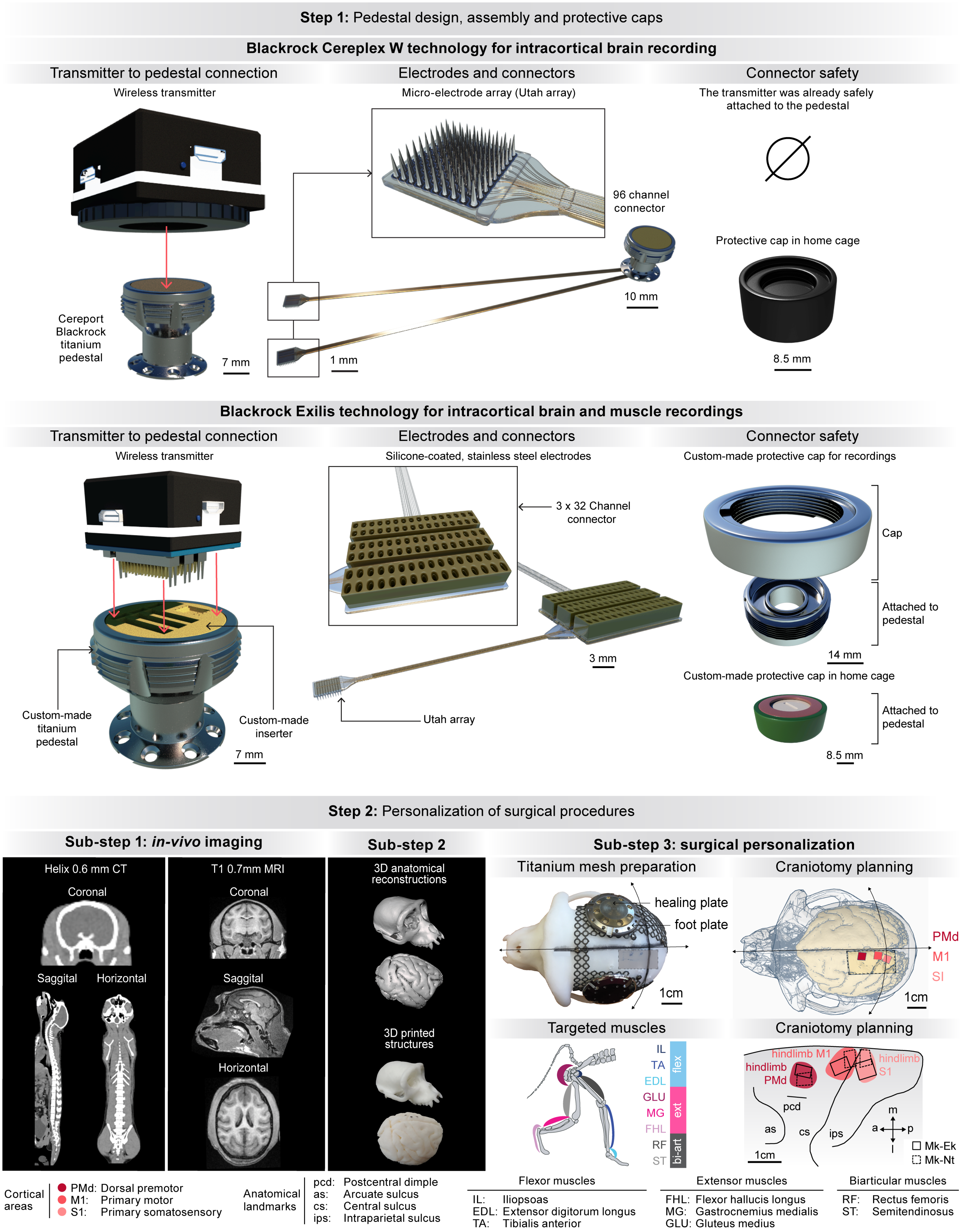

Supplementary Figure 1 | Personalized protocol for surgical implantation and fixation of custom-built skull mounted pedestals for long term percutaneous recording of electrophysiological signals. Step 1: We used two percutaneous assemblies for wireless neural and muscle activity recordings. The first was a Blackrock assembly comprising a wireless Cereplex W transmitter, a Cereport pedestal embedding the 96-channel connector connected to two Utah arrays, and a 3D printed cap to protect the pedestal in the monkeys' home cage (top panel). During recordings, the Cereplex W was safely screwed on the Cereport pedestal, thus preventing disconnections. This assembly was used to record only the neural activity. The second assembly used the Blackrock Exilis technology to record both muscle and neural activity using a single wireless transmitter (bottom panel). A 64-electrode Utah array was connected to two 32-channel connectors. 32 silicone-coated stainless-steel electrodes were connected to the third

32-channel connector. These three 32-channel connectors were then embedded in a custom-made titanium pedestal. During recordings, the Cereplex Exilis wireless transmitter was plugged in the 3x32 channel connector. However, this made the assembly fragile and prone to disconnections. We therefore designed a protective cap that was screwed onto the pedestal and protected the Exilis transmitter during the recordings. We also designed a cap that protected the pedestal and the connector while the monkeys were in their home cage. **Step 2:** The second step comprised a three-part personalized surgical procedure. Part 1: We performed structural Magnetic Resonance Imaging (MRI) and computer tomography (CT) head scans of the monkeys before the surgeries. We used the CT and MRI scans to reconstruct the skull and brain anatomy, respectively. Part 2: We co-referenced the brain anatomy to the Paxinos brain atlas to determine the location of the hindlimb areas of PMd, M1 and S1. To assist with the anatomical localization during surgery, we 3D printed the skull and brain using the anatomical reconstructions, and then marked the target areas on the prints. We used these marked prints during the surgery to orient the implantation. Part 3: We molded a titanium mesh based on the shape of the skull (Mk-Ek shown as an example). We then fixed the foot and healing plates to the mesh, allowing robust attachment of the pedestals during the surgery. We co-registered the brain and skull to plan the craniotomy and positioning of Utah arrays (Mk-Ek shown as an example). We prepared the bipolar steel electrodes according to the target hindlimb muscles. We confirmed the correct placement of the Utah arrays post-mortem (**Supplementary Fig. 2**). PMd = dorsal premotor cortex (F2), M1 = primary motor cortex (F1) and S1 = primary somatosensory cortex (area 1), IL = iliopsoas, EDL = extensor digitorum longus, TA = tibialis anterior, MG = medial gastrocnemius, FHL = flexor hallucis longus, GLU = gluteus medius, RF = rectus femoris, and ST = semitendinosus.

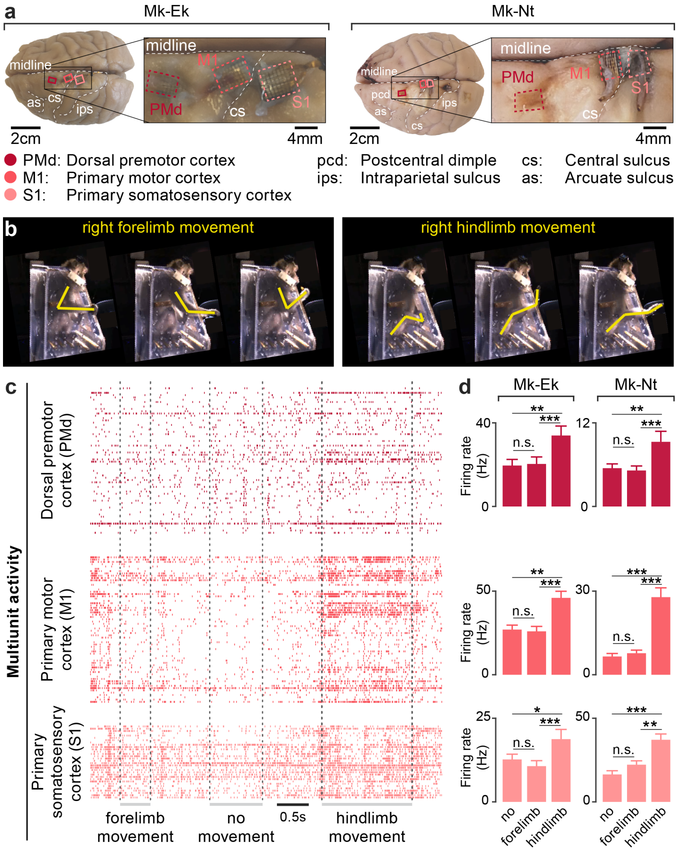

Supplementary Figure 2 | Neural recordings originate from PMd, M1 and S1 hindlimb areas. a. We performed post-mortem anatomical localization of implanted locations for both monkeys. Photographs show Mk-Ek's and Mk-Nt's brains with the implanted Blackrock microelectrode arrays. Brains were extracted after the animals were euthanized. The photographs were referenced with the macaque Paxinos brain atlas to identify cortical regions. This analysis determined that all three implanted locations in both monkeys predominantly reside in the hindlimb areas of PMd, M1 and S1, respectively. **b.** Both monkeys performed object reach-and-grasp tasks using either their right forelimb or their right hindlimb while seated in the primate chair. The photographs show Mk-Nt while she performed forelimb movement (left panel) or hindlimb movement (right panel). We used the videos to label forelimb movement, hindlimb movement and no movement epochs. **c**. Raster plot shows example multiunit activity of PMd, M1 and S1 neural populations recorded from Mk-Nt during consecutive forelimb and hindlimb movements. **d.** Neural population activity of each cortical region of both monkeys was tuned to hindlimb movements (HMs), while it remained unchanged between no movement (NM) and forelimb movement (FM) epochs. Mean firing rates of PMd (Mk-Ek: NM: 19.6±3.0; FM: 20.3±3.4; HM: 34.0±4.5; Mk-Nt: NM: 3.0±0.6; FM: 3.4±0.7; HM: 4.5±1.5), M1 (Mk-Ek: NM: 27.00±2.7; FM: 25.9±3.0; HM: 46.0±4.0; Mk-Nt: NM: 6.1±0.9; FM: 7.7±1.1; HM: 27.8±3.4) and S1 (Mk-Ek: NM: 12.7±1.6; FM: 10.7±1.7; HM: 18.7±2.9; Mk-Nt: NM: 16.1±2.4; FM: 22.2±2.3; HM: 37.1±3.5) were approximately 50% higher during hindlimb movement compared to either forelimb movement or no movement periods (PMd: Mk-Ek: HM vs. FM: $p=0.0004$; HM vs. NM: $p=0.001$; Mk-Nt: HM vs. FM: $p=0.0002$; HM vs. NM: $p=0.009$; M1: Mk-Ek: HM vs. FM: $p=0.0002$; HM vs. NM: $p=0.002$; Mk-Nt: HM vs. FM: $p=0.00000002$; HM vs. NM: $p=0.00000000003$; S1: Mk-Ek: HM vs. FM: $p=0.0004$; HM vs. NM: $p=0.034$; Mk-Nt: HM vs. FM: $p=0.005$; HM vs. NM: $p=0.0000009$). Mean firing rates during forelimb movement and no movement periods were not statistically different (PMd: Mk-Ek: FM vs. NM: $p=1$; Mk-Nt: FM vs. NM: $p=0.83$; M1: Mk-Ek: FM vs. NM: $p=1$; Mk-Nt: FM vs. NM: $p=1$; S1: Mk-Ek: FM vs. NM: $p=0.57$; Mk-Nt: FM vs. NM: $p=0.15$). The bar plot shows the mean PMd, M1 and S1 multiunit firing rate during forelimb movement, hindlimb movement and no movement epochs. Error bars: s.e.m.; n.s. $p \geq 0.05$; ** $p<0.01$; *** $p<0.001$; Kruskal–Wallis test with Bonferroni correction.

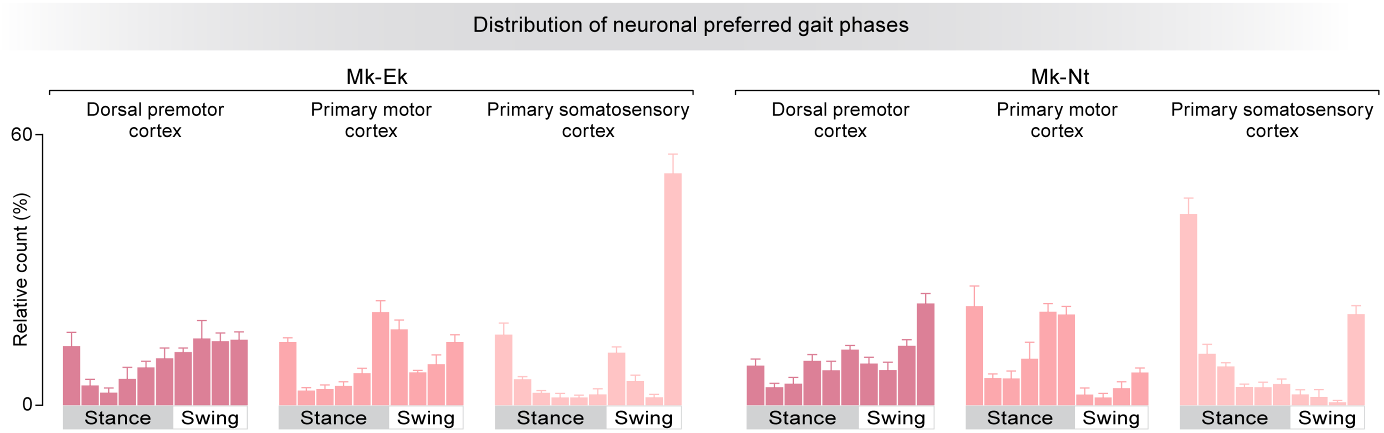

Supplementary Figure 3 | Distribution of neuronal preferred gait phases varies across sensorimotor cortical regions. Preferred gait phases are distributed largely homogeneously in PMd, bi-modally in M1 with peaks coinciding with the foot off and foot strike gait events, and unimodally in S1 with a clear peak around the foot strike event, likely reflecting the salient sensory stimulus of a foot connecting with the ground. Histograms show the distribution of preferred gait phases of isolated neurons from each cortical region. The histograms were calculated for each session and task separately, and then averaged, first across tasks and then across sessions. Error bars: s.e.m.

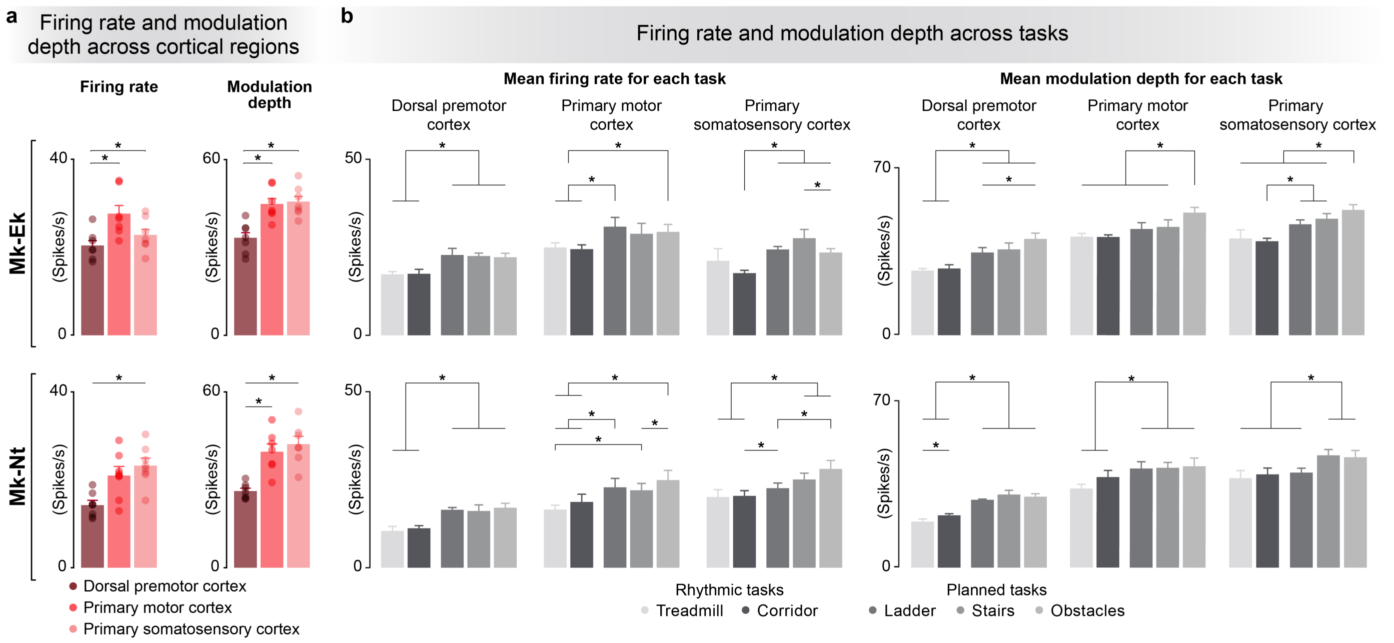

Supplementary Figure 4 | Firing rate and modulation depth are lower in PMd compared to M1 and S1, and lower for rhythmic compared to planned tasks. a. PMd shows lower neural firing rate and modulation depth compared to M1 and S1. Bars show mean firing rate and modulation depth across isolated neurons, tasks and sessions (Mk-Ek: firing rate: PMd: 20.5±1.3; M1: 27.7±2.0; S1: 22.9±1.4; M1 vs. PMd: p=0.016; PMd vs S1: p=0.031; modulation depth: PMd: 33.3±2.0; M1: 44.8±2.1; S1: 45.7±2.0; PMd vs M1: p= 0.016; PMd vs. S1: p=0.016; Mk-Nt: firing rate: PMd: 14.2±1.1; M1: 21.0±2.1; S1: 23.2±1.8; PMd vs. S1: p=0.031; modulation depth: PMd: 26.2±1.0; M1: 39.6± 2.7; S1: 42.2±2.7; PMd vs. M1: p=0.016; PMd vs. S1: p=0.016). Dots show values for each session. **b.** Bars show mean firing rate and modulation depth across isolated neurons and sessions (Mk-Ek: firing rate: PMd: treadmill: 17.3±1.0; corridor: 17.5±1.5; ladder: 22.8±2.1; stairs: 22.5±1.0; obstacles: 22.2±1.4; M1: treadmill: 25.9±1.5; corridor: 24.5±1.4; ladder: 30.9±2.8; stairs: 28.8±3.2; obstacles: 29.4±2.3; S1: treadmill: 21.1±3.7; corridor: 17.6±1.0; ladder: 24.3±1.0; stairs: 27.6±2.7; obstacles: 23.5±1.4; Mk-Ek: modulation depth: PMd: treadmill: 27.2±1.1; corridor: 28.0±1.9; ladder: 34.8±2.3; stairs: 36.0±3.0; obstacles: 40.4±2.7; M1: treadmill: 41.4±1.6; corridor: 41.2±1.2; ladder: 44.6±2.9; stairs: 45.5±3.3; obstacles: 51.5±2.5; S1: treadmill: 40.7±3.9; corridor: 39.5±1.5; ladder: 46.6±2.1; stairs: 48.9±2.5; obstacles: 52.6±2.5; Mk-Nt: firing rate: PMd: treadmill: 10.5±1.4; corridor: 11.2±0.9; ladder: 16.4±0.8; stairs: 16.1±1.9; obstacles: 16.4±1.5; M1: treadmill: 16.5±1.4; corridor: 18.7±2.4; ladder: 22.8±2.7; stairs: 21.0±2.1; obstacles: 24.9±3.0; S1: treadmill: 20.1±2.3; corridor: 20.4±1.7; ladder: 22.6±1.7; stairs: 25.1±2.0; obstacles: 28.0±2.6; modulation depth: PMd: treadmill: 19.4±1.4; corridor: 22.1±0.9; ladder: 28.6±0.6; stairs: 30.8±2.1; obstacles: 29.9±1.5; M1: treadmill: 33.3±2.1; corridor: 38.2±3.1; ladder: 41.8±3.2; stairs: 42.1±2.6; obstacles: 42.7±3.8; S1: treadmill: 37.7±3.5; corridor: 39.3±2.9; ladder: 40.0±2.1; stairs: 47.3±2.7; obstacles: 46.5±3.1). Rhythmic tasks of treadmill and corridor tended to exhibit lower neural firing rate and modulation depth compared to planned tasks of ladder, stairs and obstacles (Mk-Ek: firing rate: PMd: treadmill vs. corridor: p=0.94; treadmill vs. ladder: p=0.016; treadmill vs. stairs: p=0.031; treadmill vs. obstacles: p=0.016; corridor vs. ladder: p=0.016; corridor vs. stairs: p=0.016; corridor vs. obstacles: p=0.016; ladder vs. stairs: p=0.81; ladder vs. obstacles: p=0.69; stairs vs. obstacles: p=0.81; M1: treadmill vs. corridor: p=0.81; treadmill vs. ladder: p=0.031; treadmill vs. stairs: p=0.16; treadmill vs. obstacles: p=0.078; corridor vs. ladder: p=0.016; corridor vs. stairs: p=0.11; corridor vs. obstacles: p=0.016; ladder vs. stairs: p=0.16; ladder vs. obstacles: p=0.30; stairs vs. obstacles: p=0.94; S1: treadmill vs. corridor: p=0.38; treadmill vs. ladder: p=0.30; treadmill vs. stairs: p=0.22; treadmill vs. obstacles: p=0.30; corridor vs. ladder: p=0.016; corridor vs. stairs: p=0.016; corridor vs. obstacles: p=0.016; ladder vs. stairs: p=0.11; ladder vs. obstacles: p=0.30; stairs vs. obstacles: p=0.016; modulation depth: PMd: treadmill vs. corridor: p=0.58; treadmill vs. ladder: p=0.016; treadmill vs. stairs: p=0.016; treadmill vs. obstacles: p=0.016; corridor vs. ladder: p=0.016; corridor vs. stairs: p=0.016; corridor vs. obstacles: p=0.016; ladder vs. stairs: p=0.81; ladder vs. obstacles: p=0.016; stairs vs. obstacles: p=0.16; M1: treadmill vs. corridor: p=0.81; treadmill vs. ladder: p=0.22; treadmill vs. stairs: p=0.16; treadmill vs. obstacles: p=0.016; corridor vs. ladder: p=0.16; corridor vs. stairs: p=0.16; corridor vs. obstacles: p=0.016; ladder vs. stairs: p=0.58; ladder vs. obstacles: p=0.016; stairs vs. obstacles: p=0.016; S1: treadmill vs. corridor: p=0.30; treadmill vs. ladder: p=0.30; treadmill vs. stairs: p=0.22; treadmill vs. obstacles: p=0.031; corridor vs. ladder: p=0.031; corridor vs. stairs: p=0.016; corridor vs. obstacles: p=0.016; ladder vs. stairs: p=0.078; ladder vs. obstacles: p=0.016; stairs vs. obstacles: p=0.016; Mk-Nt: firing rate: PMd: treadmill vs. corridor: p=0.94; treadmill vs. ladder: p=0.016; treadmill vs. stairs: p=0.016; treadmill vs. obstacles: p=0.016; corridor vs. ladder: p=0.016; corridor vs. stairs: p=0.016; corridor vs. obstacles: p=0.016; ladder vs. stairs: p=1; ladder vs. obstacles: p=0.58; stairs vs. obstacles: p=0.81; M1: treadmill vs. corridor: p=0.94; treadmill vs. ladder: p=0.016; treadmill vs. stairs: p=0.016; treadmill vs. obstacles: p=0.016; corridor vs. ladder: p=0.016; corridor vs. stairs: p=0.078; corridor vs. obstacles: p=0.016; ladder vs. stairs: p=0.94; ladder vs. obstacles: p=0.078; stairs vs. obstacles: p=0.031; S1: treadmill vs. corridor: p=1; treadmill vs. ladder: p=0.30; treadmill vs. stairs: p=0.031; treadmill vs. obstacles: p=0.016; corridor vs. ladder: p=0.016; corridor vs. stairs: p=0.016; corridor vs. obstacles: p=0.016; ladder vs. stairs: p=0.078; ladder vs. obstacles: p=0.016; stairs vs. obstacles: p=0.078; modulation depth: PMd: treadmill vs. corridor: p=0.047; treadmill vs. ladder: p=0.016; treadmill vs. stairs: p=0.016; treadmill vs. obstacles: p=0.016; corridor vs. ladder: p=0.016; corridor vs. stairs: p=0.016; corridor vs. obstacles: p=0.016; ladder vs. stairs: p=0.30; ladder vs. obstacles: p=0.16; stairs vs. obstacles: p=0.58; M1: treadmill vs. corridor: p=0.16; treadmill vs. ladder: p=0.016; treadmill vs. stairs: p=0.016; treadmill vs. obstacles: p=0.016; corridor vs. ladder: p=0.016; corridor vs. stairs: p=0.016; corridor vs. obstacles: p=0.016; ladder vs. stairs: p=0.94; ladder vs. obstacles: p=0.69; stairs vs. obstacles: p=0.38; S1: treadmill vs. corridor: p=0.69; treadmill vs. ladder: p=0.47; treadmill vs.

stairs: $p=0.016$; treadmill vs. obstacles: $p=0.016$; corridor vs. ladder: $p=0.58$; corridor vs. stairs: $p=0.016$; corridor vs. obstacles: $p=0.016$; ladder vs. stairs: $p=0.016$; ladder vs. obstacles: $p=0.016$; stairs vs. obstacles: $p=0.94$). For visualization purposes, we only depicted the statistically significant comparisons at $p<0.05$. Error bars: s.e.m.; * $p<0.05$. Wilcoxon signed rank test.

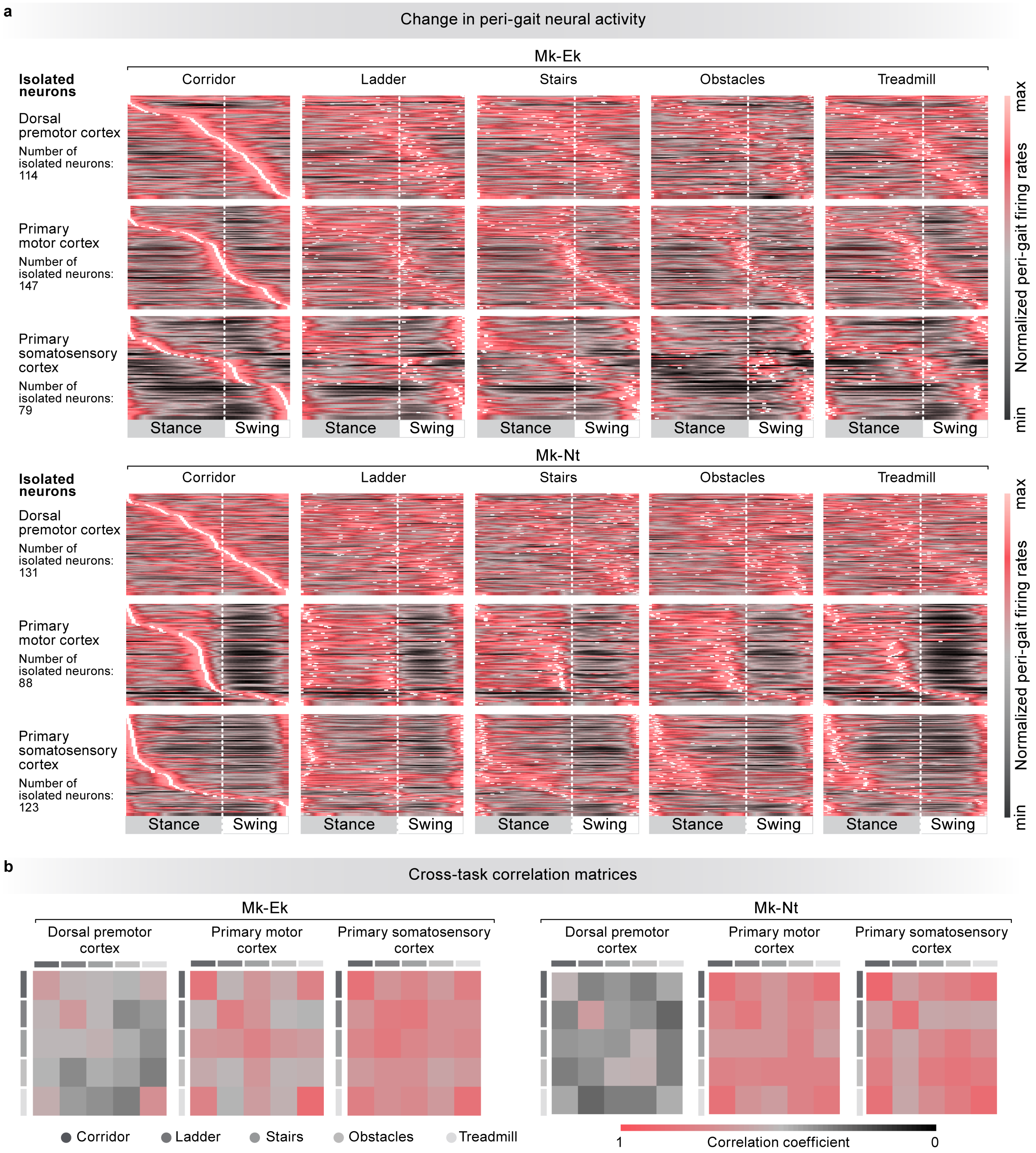

Supplementary Figure 5 | Neural population activity changes across tasks. a. The colorplots show normalized peri-gait firing rates of isolated neurons sorted by the preferred gait phase on the corridor task. Note the substantial changes in neural activity, reflected in changes of gait phase tuning of each neuron. The neurons recoded in different sessions are here grouped together for visualization purposes only. All statistics in our study are calculated by treating different sessions as separate data sets with no assumptions about whether the neurons recorded in different sessions are the same or different. **b.** For each neuron, we computed Pearson's linear correlation coefficient between the activity in any two gait cycles, either from the same or a different task. To obtain cross-task correlation, we averaged these values across all gait cycle pairs in a particular task combination (10 combinations), all neurons from a cortical region, and all sessions. The colorplots show these cross-task correlations for all task combinations and for both monkeys.

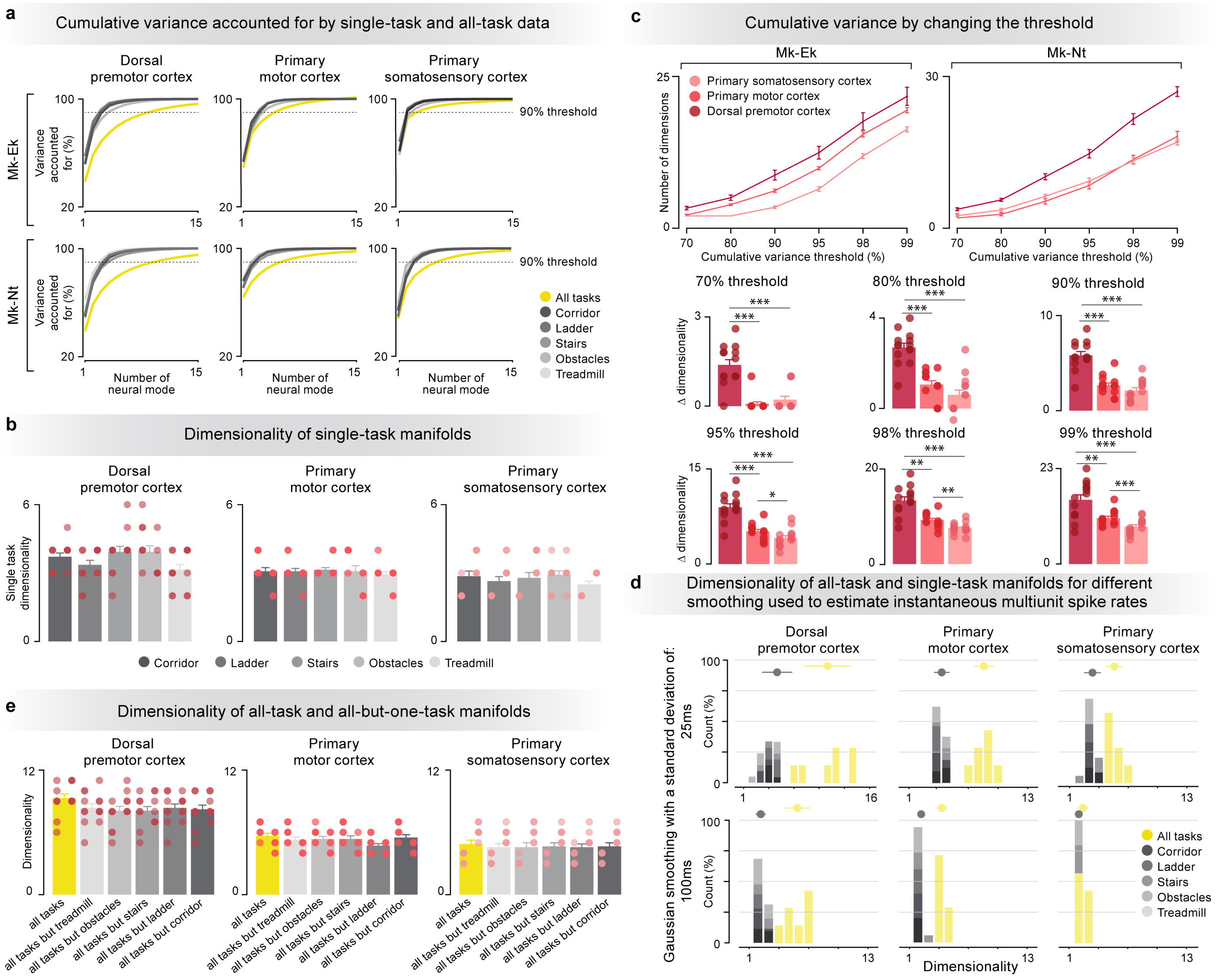

Supplementary Figure 6 | Variance explained by neural manifolds and contribution of each task towards the dimensionality of all-task manifolds. **a**. The dimensionality of a dataset was measured to be the number of modes needed to explain more than 90% of the cumulative variance. The plot shows the mean cumulative variance explained by neural modes for single-tasks and all-task datasets across all sessions. **b**. The bars show the mean dimensionality of single-task manifolds across sessions and monkeys. Dots show values for each session and monkey (Mk-Ek: left column; Mk-Nt: right column). **c.** PMd all-task manifold dimensionality remains substantially higher than M1 and S1 all-task manifolds across a wide range of thresholds used to determine the all-task manifold (70% threshold: PMd vs. M1: p=0.0005; PM vs. S1: p=0.0005; M1 vs. S1: p=0.63; 80% threshold: PMd vs. M1: p=0.0004; PM vs. S1: p=0.0001; M1 vs. S1: p=0.15; 90% threshold: PMd vs. M1: p=0.0002; PM vs. S1: p=0.0001; M1 vs. S1: p=0.15; 95% threshold: PMd vs. M1: p=0.0005; PM vs. S1: p=0.0001; M1 vs. S1: p=0.045; 98% threshold: PMd vs. M1: p=0.002; PM vs. S1: p=0.0001; M1 vs. S1: p=0.002; 99% threshold: PMd vs. M1: p=0.002; PM vs. S1: p=0.0001; M1 vs. S1: p=0.0005). The plots show the mean all-task manifold dimensionality across all sessions for each cortical region and each monkey. The bar plots show the mean difference (Δ) in dimensionality between the all-task and single-task manifolds across sessions and monkeys (same analysis as shown in **Fig. 4d**) for different thresholds used to determine the manifolds. **d.** The bars show the histogram of single-task (greyscale-coded) and all-task (yellow) manifold dimensionality across all sessions for Mk-Ek for using Gaussian smoothing with a standard deviation of 25ms and 100ms when estimating multiunit spike rates. This analysis replicates the one in **Fig. 4b** for different Gaussian smoothing lengths (there shown for Gaussian smoothing with a standard deviation of 50ms). The dots show the mean dimensionality of single-task (gray) and all-task (yellow) manifolds. **e.** The bars show the mean dimensionality of all-task and all-but-one-task manifolds across sessions and monkeys. Dots show values for each session and monkey (Mk-Ek: left column; Mk-Nt: right column). Error bars: s.e.m.; * p<0.05; ** p<0.01; *** p<0.001; Wilcoxon signed rank test.

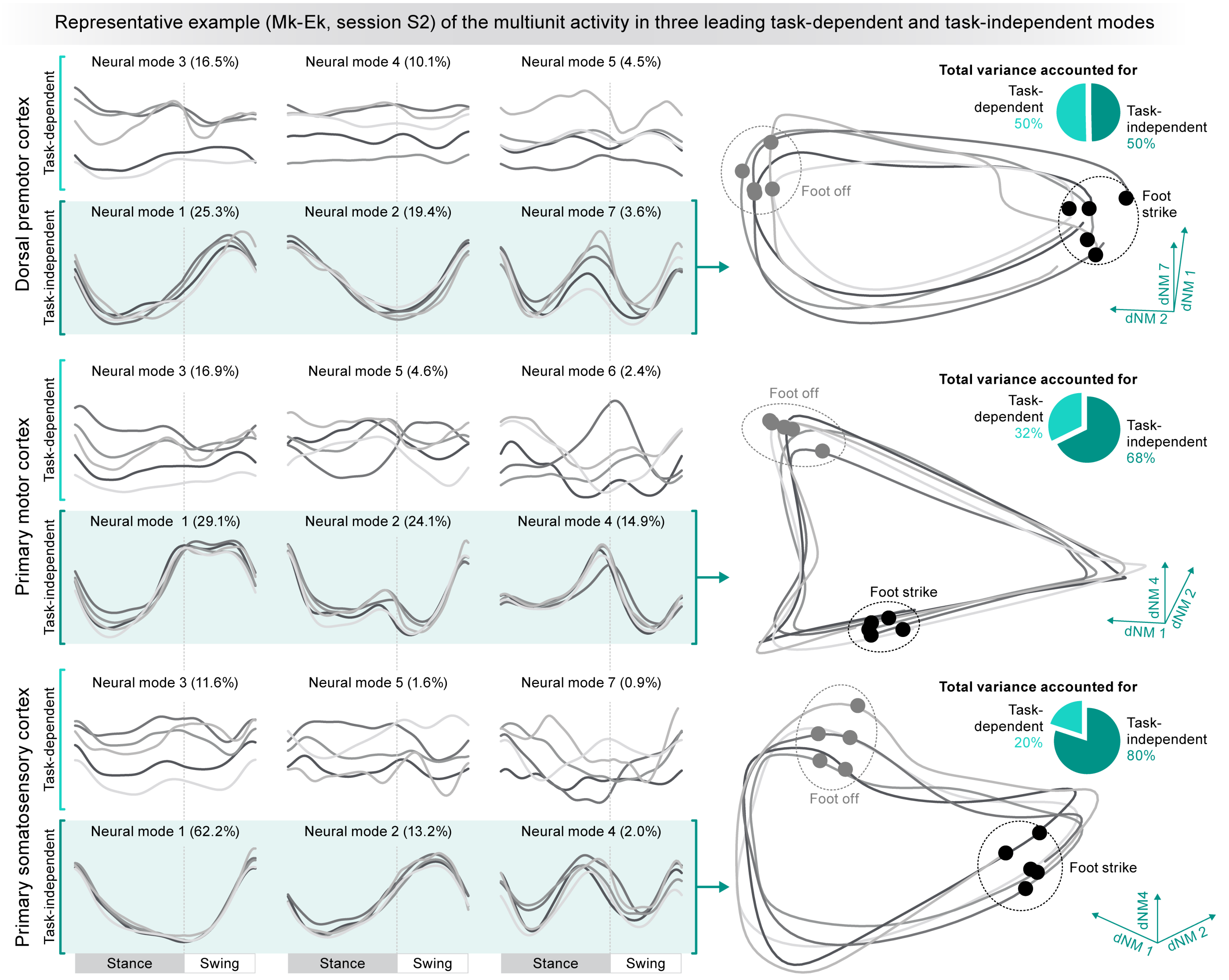

Supplementary Figure 7 | Activity in three leading task-independent modes of PMd, M1 and S1 traces similar trajectories for all tasks. The 2D plots show the peri-gait activity of three task-dependent and task-independent modes that explained the highest amounts of neural population activity variance for a session S2 in Mk-Ek. The 3D plots show projection of peri-gait neural population activity of a cortical region into a space spanned by the three leading task-independent modes, here depicted by their corresponding demixed principal component (dPC) numbers. The pie plots show the total amount of neural variance accounted for (VAF) by the task independent (blue) or task-dependent (red) subspaces for session S2.

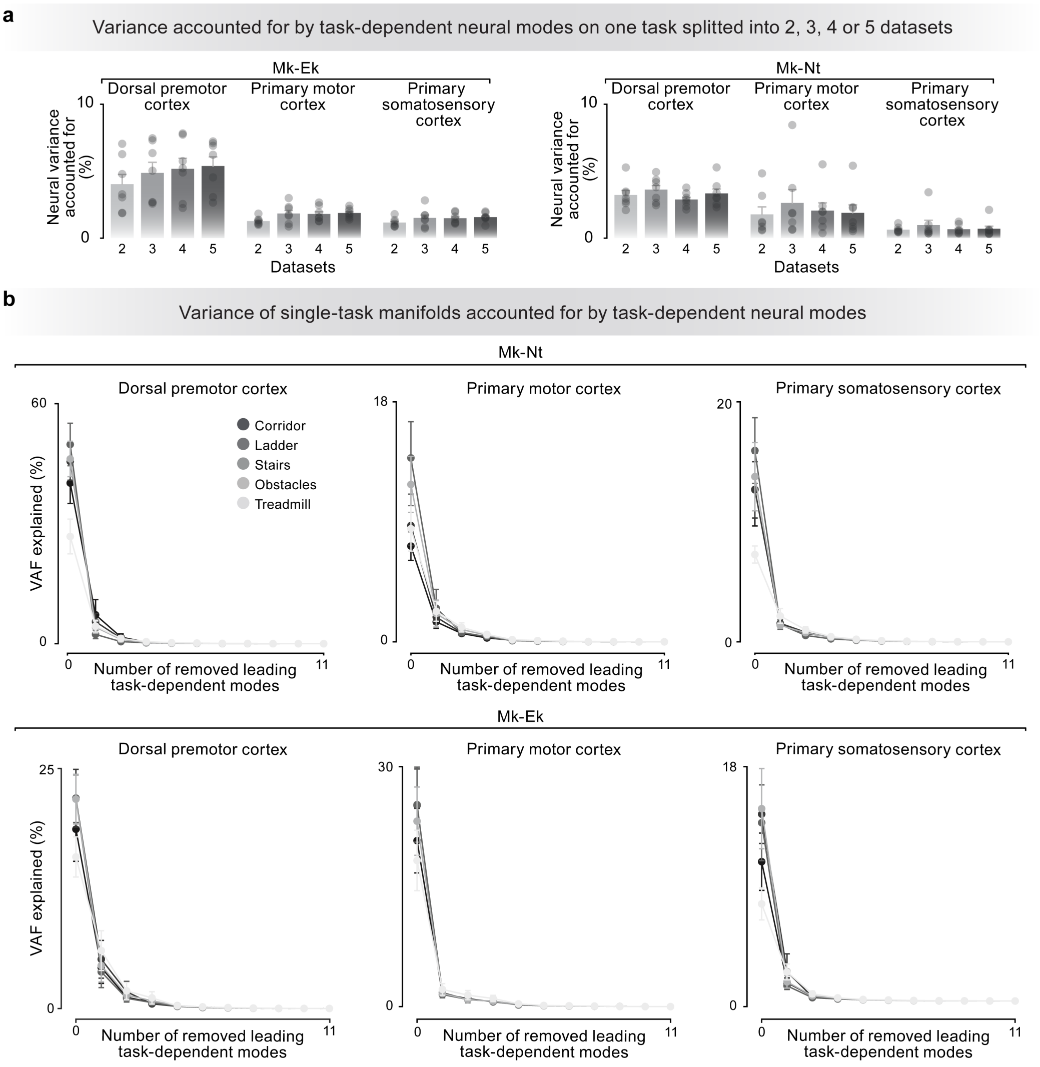

Supplementary Figure 8 | Detailed evaluation of demixed principal component analysis results. a. Within-task behavioral variability accounts for less than 6% of the task-dependent variance across all tasks and all three cortical regions. Some of the variance in the task-dependent subspaces may come from task-unrelated sparse behavioral events that were arbitrarily assigned to a specific task (e.g., monkey stopping to stretch). Despite being task-unrelated, this behavior will be assigned to the task-dependent variance. To quantify the amount of such variance that may be attributed to task-dependent subspace, for each session and task, we created surrogate datasets in which trials of the same task were randomly distributed into 2, 3, 4 or 5 randomly selected datasets forming surrogate tasks. This will assign the sparse behavioral events to one of the surrogate tasks. We then computed the total variance accounted for (VAF) by the surrogate-task-dependent neural modes. Note that the standard task-related behavior will now occupy the surrogate-task-independent subspace. Thus, the neural variance accounted for by the surrogate-task-dependent subspace estimates the amount of variance that may come from the behavioral variability. We performed this analysis for each cortical region separately. The bars show mean neural VAF across sessions. Dots show VAF for each session. **b.** Removing leading task-dependent modes reduces similar amount of variance from all single-task manifolds. The plots show the mean portion of lost single-task manifold variance when sequentially removing leading task-dependent neural modes. Errorbars: s.e.m.

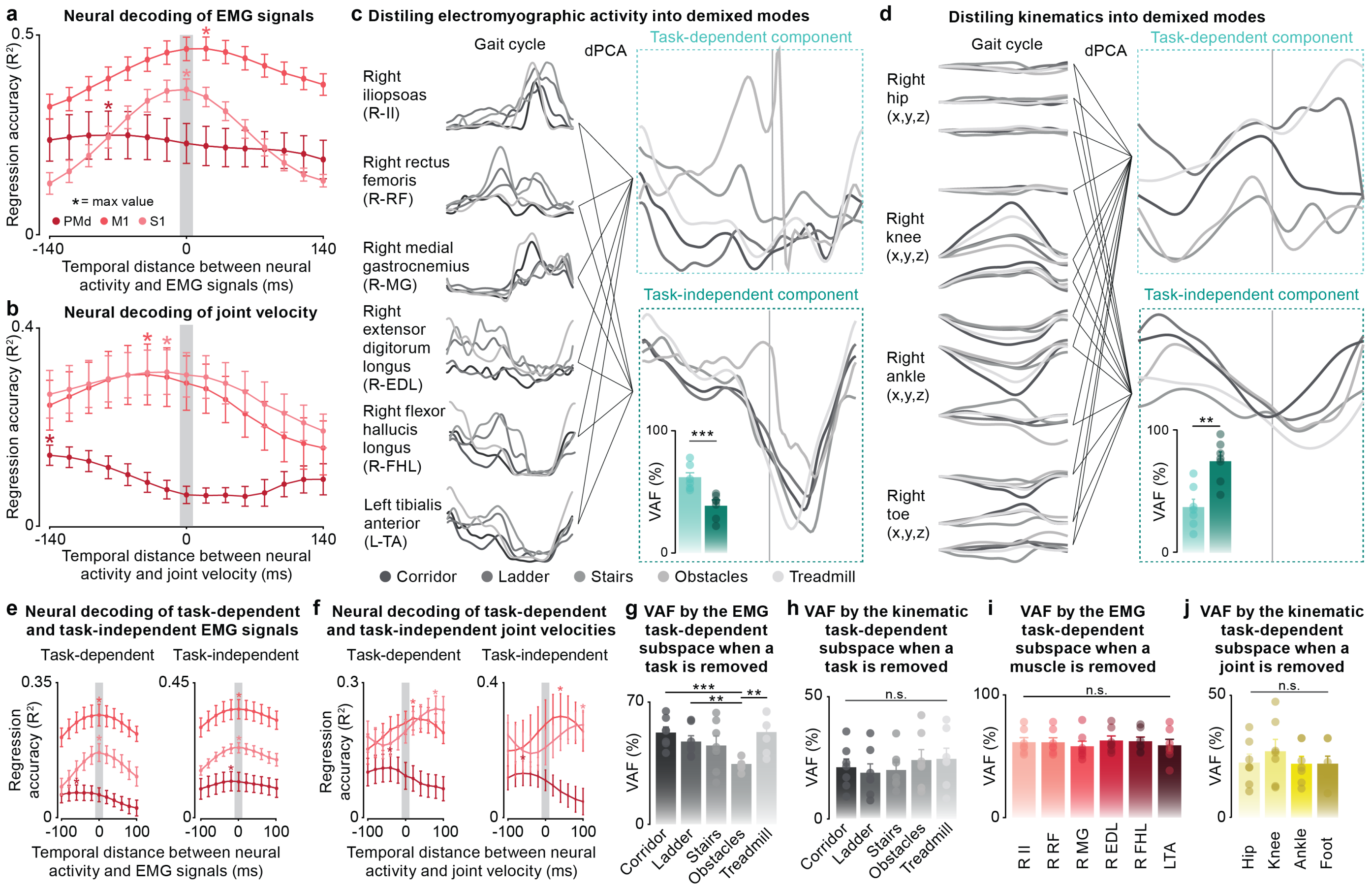

Supplementary Figure 9 | PMd population activity poorly predicts hindlimb electromyography (EMG) and kinematics. a. PMd explains the least hindlimb EMG variance when compared to M1 and S1. The graph shows the mean variance explained for (R^2) by EMG envelopes reconstructed by a Wiener filter decoder applied on the PMd, M1 and S1 neural population activity. We used the Wiener filter to decode EMG at different temporal lags in the future or past with respect to the neural population activity. The stars indicate the temporal lags of maximum R^2 for each cortical region. **b.** PMd explains the least hindlimb kinematic (joint velocity in x,y,z coordinates) variance when compared to M1 and S1. The graph shows the mean variance explained for (R^2) by kinematics reconstructed by a Wiener filter decoder applied on the PMd, M1 and S1 neural population activity. The Wiener filter was used to decode kinematics at different temporal lags in the future or the past with respect to the neural population activity. **c.** Majority of EMG envelope variance is explained by its task-dependent modes. We applied dPCA to hindlimb EMGs in Mk-Ek to derive task-dependent and task-independent neural modes. Panels show the mean peri-gait EMG envelopes and the leading task-dependent and task-independent neural modes. Bar plots show the mean EMG envelope variance accounted for (VAF) by the task-dependent and test-independent subspaces across all seven sessions (p=0.0006). Dots show values for individual sessions. **d.** Majority of hindlimb kinematics is explained by its task-independent modes. We applied dPCA to right hindlimb kinematic in Mk-Nt to derive task-dependent and task-independent neural modes. Panels show the mean peri-gait joint coordinates and its leading task-dependent and task-independent neural modes. Bar plots show the mean kinematic variance accounted for (VAF) by the task-dependent and test-independent subspaces across all seven sessions (p=0.004). Dots show values for individual sessions. **e.** Reconstruction of task-dependent and task-independent EMG subspaces from neural population activity qualitatively reflects the reconstruction of the complete EMG envelopes (see **a**): PMd explains the least variance when compared to S1 and M1. The graph shows the mean variance explained for (R^2) by EMG envelopes inferred by a Wiener filter algorithm applied on the PMd, M1 and S1 neural population activity. **f.** Reconstruction of task-dependent and task-independent kinematic subspaces from neural population activity qualitatively reflects the reconstruction of the complete kinematic (see **b**): PMd explains the least variance when compared to S1 and M1. The graph shows the mean variance explained for (R^2) by kinematics inferred by a Wiener filter algorithm applied on the PMd, M1 and S1 neural population activity. **g.** None of the tasks have a dominant influence on the EMG envelope task-dependent subspace. The bars show the variance accounted for (VAF) by the EMG task-dependent subspace when one task is removed (corridor vs. obstacles: p=0.0006; ladder vs. obstacles: p=0.007; obstacles vs. treadmill: p=0.002). Dots show values for individual sessions. **h.** None of the tasks have a dominant influence on the kinematic task-dependent subspace. The bars show the variance accounted for (VAF) by the kinematic task-dependent subspace when one task is removed. Dots show values for individual sessions. **i.** None of the muscles dominate the EMG envelope task-dependent subspace. The bars show the variance accounted for (VAF) by the EMG task-dependent subspace when one muscle is removed. Dots show values for individual sessions. **j.** None of the joints dominate the kinematic task-dependent subspace. The bars show the variance accounted for (VAF) by the kinematic task-dependent subspace when one joint is removed. Dots show values for individual sessions. Error bars: s.e.m.; n.s. p≥0.05; * p<0.05; ** p<0.01; *** p<0.001. Wilcoxon signed rank test.

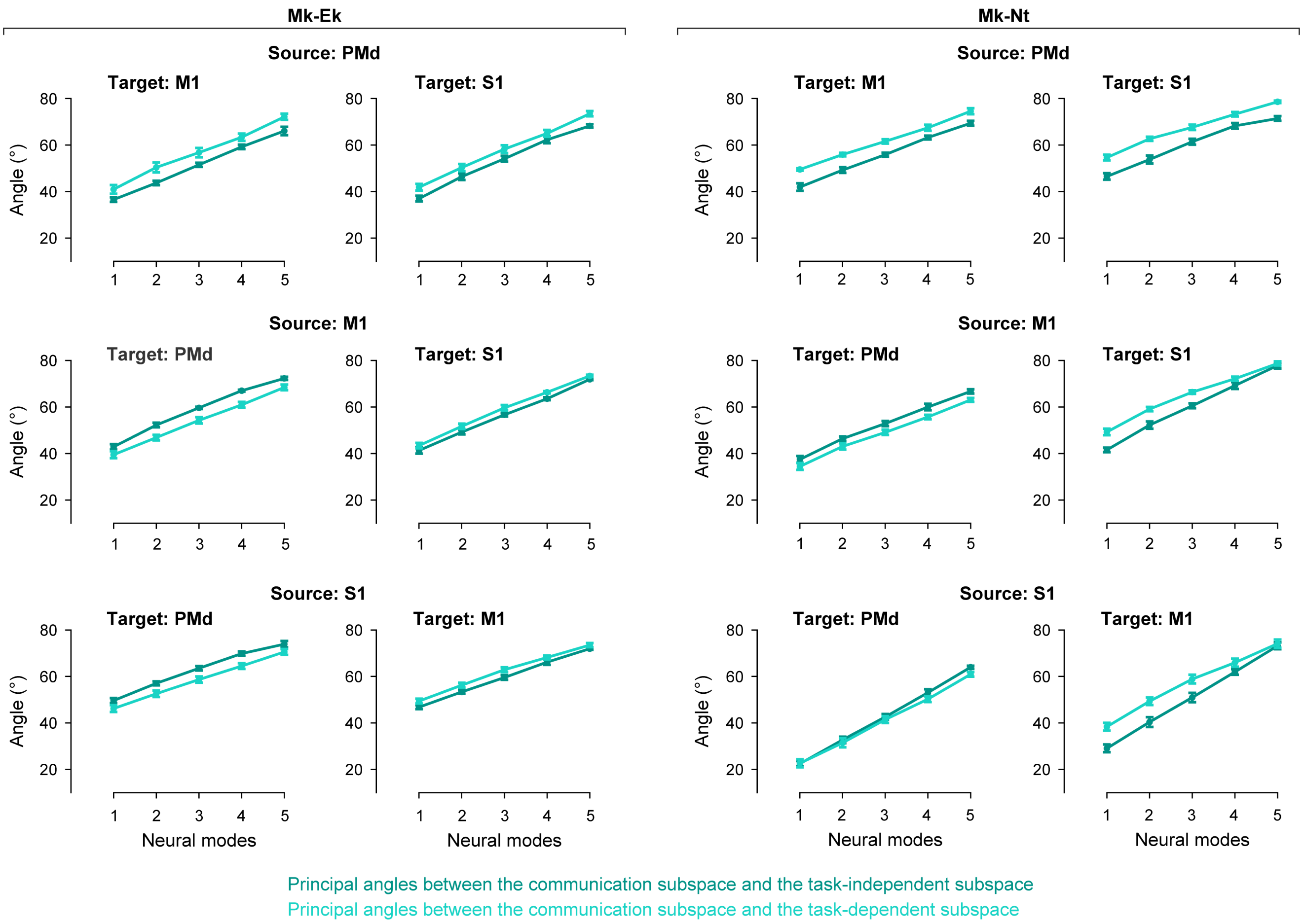

Supplementary Figure 10 | Principal angles between the communication subspace and the task-dependent or task-independent subspaces. The panels show mean of all five principal angles across tasks and sessions for each source and target cortical region and each monkey. Error bars: s.e.m.

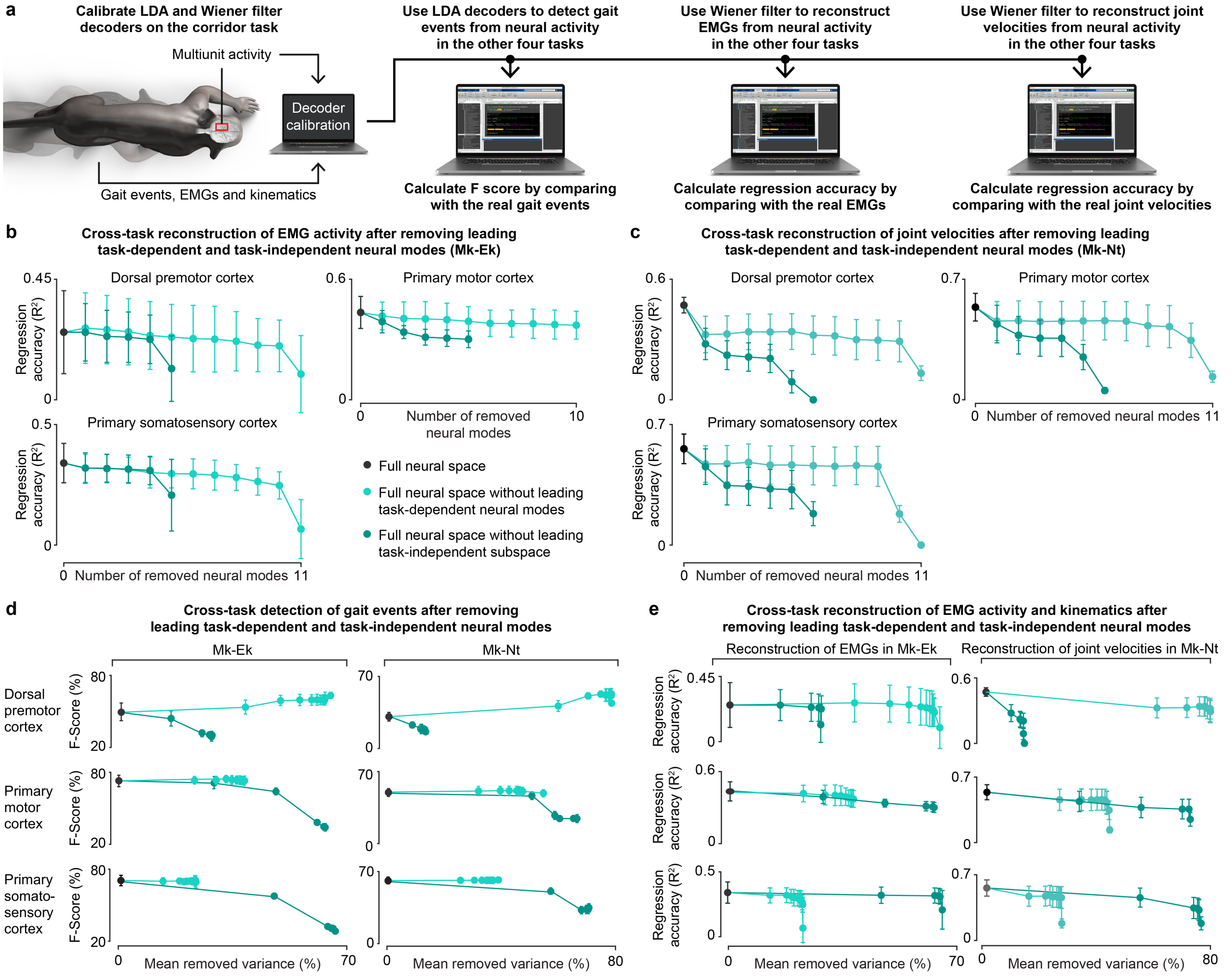

Supplementary Figure 11 | Cross-task decoding of gait events, muscle activity and kinematics deteriorates when removing leading task-independent neural modes, but remains as accurate when removing task-dependent neural modes. a. Illustration shows the conceptual design of the decoding analysis. We calibrated decoders using data collected in the corridor task to detect right hindlimb gait events, or to reconstruct right hindlimb EMG envelopes, or to reconstruct right hindlimb join velocities from the neural population activity from one of the three cortices. We then quantified the accuracy of these decoder when applied on the data from the other four tasks: ladder, obstacles, staircase and treadmill. **b.** Sequentially subtracting leading task-dependent or task-independent neural modes doesn't substantially deteriorate the reconstruction of EMG envelopes from PMd, M1 and S1 neural population activity. The plots show regression accuracy (R^2) of a Wiener filter algorithm applied on the PMd, M1 and S1 neural population activity of Mk-Ek. **c**. Sequentially removing leading task-independent neural modes deteriorates reconstruction of kinematics from PMd, M1 and S1 neural population activity more when compared to sequential removal of leading task-dependent neural modes. The plots show regression accuracy (R^2) of a Wiener filter algorithm applied on the PMd, M1 and S1 neural population activity of Mk-Nt. **d**. The plots show the accuracy of detecting gait events (F-score) for datasets containing all neural activity (black dot) or the same datasets with leading task-dependent (light green lines) or task-independent modes (dark green lines) removed as a function of mean removed neural population activity variance. **e.** The plots show regression accuracy (R^2) of a Wiener filter algorithm applied on the PMd, M1 and S1 neural population activity that contains all neural activity (black dot) or the same datasets with leading task-dependent (light green lines) or task-independent modes (dark green lines) removed as a function of removed neural population activity variance. Errorbars: s.e.m.

Timing
Step duration in seconds
Stance duration in seconds
Swing duration in seconds
Foot-off timing
Duration of stance in percentage of gait cycle
Duration from foot strike to maximal foot elevation
Duration from maximal foot elevation to foot strike

Step dimension
Maximal foot elevation
Length of step
Length of stride
foot-off timing
Path length of ankle marker

Posture
Lateral displacement during swing
Lateral displacement during stance

Joint angles
Maximal hip angle
Minimal hip angle
Maximal knee angle
Minimal knee angle
Maximal ankle angle
Minimal ankle angle
Maximal limb axis angle
Minimal limb axis angle

Forward-backward elevation angles
Maximal trunk elevation angle
Minimal trunk elevation angle
Maximal thight elevation angle
Minimal thight elevation angle
Maximal shank elevation angle
Minimal shank elevation angle
Maximal foot elevation angle
Minimal foot elevation angle
Maximal limb elevation angle
Minimal limb elevation angle

Oscillation amplitude
Difference hip angle
Difference knee angle
Difference ankle angle
Difference limb axis
Limb length
Difference trunk elevation angle
Difference thigh elevation angle
Difference shank elevation angle
Difference foot elevation angle
Difference limb elevation angle

Joint oscillatory velocities
Maximal speed hip angle
Minimal speed hip angle
Maximal speed knee angle
Minimal speed knee angle
Maximal speed ankle angle
Minimal speed ankle angle
Maximal speed limb elevation angle
Minimal speed limb elevation angle
Difference speed hip angle
Difference speed knee angle
Difference speed ankle angle
Difference speed limb elevation angle

Endpoint control
Maximal speed of foot
Acceleration of foot at swing onset
Speed of foot at swing onset
Time of maximal speed of foot in percentage of gait cycle

Supplementary Table 1 | Kinematic features computed
