# Supplementary material for: Regional specialization of movement encoding across the primate sensorimotor cortex
Source: Nat Commun. 2025 Jul 1;16:5729. doi: 10.1038/s41467-025-61172-8 (PMC12216890; doi:10.1038/s41467-025-61172-8)
Supplement: Supplementary file 2 — Reporting Summary [file 41467_2025_61172_MOESM2_ESM.pdf]

nature portfolio

Corresponding author(s): Grégoire Courtine, PhD; Ismael Seáñez, PhD; Tomislav Milekovic, PhD

Last updated by author(s): May 28, 2025

Reporting Summary

Nature Portfolio wishes to improve the reproducibility of the work that we publish. This form provides structure for consistency and transparency in reporting. For further information on Nature Portfolio policies, see our Editorial Policies and the Editorial Policy Checklist.

Statistics

For all statistical analyses, confirm that the following items are present in the figure legend, table legend, main text, or Methods section.

n/a	Confirmed	
☐	☒	The exact sample size (*n*) for each experimental group/condition, given as a discrete number and unit of measurement
☐	☒	A statement on whether measurements were taken from distinct samples or whether the same sample was measured repeatedly
☐	☒	The statistical test(s) used AND whether they are one- or two-sided<br>*Only common tests should be described solely by name; describe more complex techniques in the Methods section.*
☐	☒	A description of all covariates tested
☐	☒	A description of any assumptions or corrections, such as tests of normality and adjustment for multiple comparisons
☐	☒	A full description of the statistical parameters including central tendency (e.g. means) or other basic estimates (e.g. regression coefficient) AND variation (e.g. standard deviation) or associated estimates of uncertainty (e.g. confidence intervals)
☐	☒	For null hypothesis testing, the test statistic (e.g. *F*, *t*, *r*) with confidence intervals, effect sizes, degrees of freedom and *P* value noted<br>*Give P values as exact values whenever suitable.*
☒	☐	For Bayesian analysis, information on the choice of priors and Markov chain Monte Carlo settings
☒	☐	For hierarchical and complex designs, identification of the appropriate level for tests and full reporting of outcomes
☐	☒	Estimates of effect sizes (e.g. Cohen's *d*, Pearson's *r*), indicating how they were calculated

Our web collection on statistics for biologists contains articles on many of the points above.

Software and code

Policy information about availability of computer code

Data collection	All software used for data collection is described in the Method section at the relevant paragraph. Following is a list of softwares used: Simi Motion software by Simi Reality Motion Systems, Cerebus software suite by Blackrock Microsystems, and 3 Tesla GE Medical system Discovery MR750 software.
Data analysis	All software used for data analysis is described in the Method section at the relevant paragraph. Following is a list of softwares used: Simi Motion software by Simi Reality Motion Systems, FSL v.5.0.9, OsiriX v.3.0.2, Blender v.2.78, Matlab v2018a and later by Mathworks, Meshmixer by Autodesk Inc., and Offline Sorter by Plexon Inc.

For manuscripts utilizing custom algorithms or software that are central to the research but not yet described in published literature, software must be made available to editors and reviewers. We strongly encourage code deposition in a community repository (e.g. GitHub). See the Nature Portfolio guidelines for submitting code & software for further information.

Data

Policy information about availability of data

All manuscripts must include a data availability statement. This statement should provide the following information, where applicable:

- Accession codes, unique identifiers, or web links for publicly available datasets
- A description of any restrictions on data availability
- For clinical datasets or third party data, please ensure that the statement adheres to our policy

Source data are provided with this paper.

Research involving human participants, their data, or biological material

Policy information about studies with human participants or human data. See also policy information about sex, gender (identity/presentation), and sexual orientation and race, ethnicity and racism.

Reporting on sex and gender	*Use the terms sex (biological attribute) and gender (shaped by social and cultural circumstances) carefully in order to avoid confusing both terms. Indicate if findings apply to only one sex or gender; describe whether sex and gender were considered in study design; whether sex and/or gender was determined based on self-reporting or assigned and methods used. Provide in the source data disaggregated sex and gender data, where this information has been collected, and if consent has been obtained for sharing of individual-level data; provide overall numbers in this Reporting Summary. Please state if this information has not been collected. Report sex- and gender-based analyses where performed, justify reasons for lack of sex- and gender-based analysis.*
Reporting on race, ethnicity, or other socially relevant groupings	*Please specify the socially constructed or socially relevant categorization variable(s) used in your manuscript and explain why they were used. Please note that such variables should not be used as proxies for other socially constructed/relevant variables (for example, race or ethnicity should not be used as a proxy for socioeconomic status). Provide clear definitions of the relevant terms used, how they were provided (by the participants/respondents, the researchers, or third parties), and the method(s) used to classify people into the different categories (e.g. self-report, census or administrative data, social media data, etc.) Please provide details about how you controlled for confounding variables in your analyses.*
Population characteristics	*Describe the covariate-relevant population characteristics of the human research participants (e.g. age, genotypic information, past and current diagnosis and treatment categories). If you filled out the behavioural & social sciences study design questions and have nothing to add here, write "See above."*
Recruitment	*Describe how participants were recruited. Outline any potential self-selection bias or other biases that may be present and how these are likely to impact results.*
Ethics oversight	*Identify the organization(s) that approved the study protocol.*

Note that full information on the approval of the study protocol must also be provided in the manuscript.

Field-specific reporting

Please select the one below that is the best fit for your research. If you are not sure, read the appropriate sections before making your selection.

☒ Life sciences ☐ Behavioural & social sciences ☐ Ecological, evolutionary & environmental sciences

For a reference copy of the document with all sections, see nature.com/documents/nr-reporting-summary-flat.pdf

Life sciences study design

All studies must disclose on these points even when the disclosure is negative.

Sample size	Data from 2 non-human primates, Mk-Nt and Mk-Ek, recorded over 7 sessions for each monkey was used for all the analysis. Each session included five tasks: corridor, ladder, stairs, obstacles, and treadmill at 3km/h. The analyzed data included 295, 312, 124, 208, and 256 gait cycles in Mk-Nt; and 140, 197, 176, 66, and 320 gait cycles in Mk-Ek retained for analysis for corridor, ladder, stairs, obstacles, and treadmill at 3km/h, respectively. Our previous analyses indicated that collection of data from 7 sessions will be sufficient to obtain statistically-significant results in both animals.
Data exclusions	Gait cycles, defined as the epoch between two consecutive foot strikes, were rejected if the duration of the step was longer than 1.5 seconds. Additionally, we rejected gait cycles with an “outlier” stance phase duration, lasting from the right hindlimb foot strike to the right hindlimb foot off, that exceeded 1.5 times the 75th and 25th interquartile range.
Replication	The analysis of kinematic, electromyography and neurophisiological datasets of non-human primates was performed on seven sessions, each recorded on a different day. Replication numbers for analysis of human data are provided in detail in figure captions.
Randomization	We randomized the order of tasks in each session.

Blinding	Not applicable. Our experiemnts did not include group allocation.

Reporting for specific materials, systems and methods

We require information from authors about some types of materials, experimental systems and methods used in many studies. Here, indicate whether each material, system or method listed is relevant to your study. If you are not sure if a list item applies to your research, read the appropriate section before selecting a response.

Materials & experimental systems

n/a	Involved in the study
☒	☐ Antibodies
☒	☐ Eukaryotic cell lines
☒	☐ Palaeontology and archaeology
☐	☒ Animals and other organisms
☒	☐ Clinical data
☒	☐ Dual use research of concern
☒	☐ Plants

Methods

n/a	Involved in the study
☒	☐ ChIP-seq
☒	☐ Flow cytometry
☐	☒ MRI-based neuroimaging

Animals and other research organisms

Policy information about studies involving animals; ARRIVE guidelines recommended for reporting animal research, and Sex and Gender in Research

Laboratory animals	We recorded the data from 2 fascicularis macaques that were 7 and 9 years old and weighed between 3.5 and 4.6 kg .
Wild animals	None.
Reporting on sex	Both animals were female.
Field-collected samples	Not applicable.
Ethics oversight	All the experimental procedures were approved by the Federal and local veterinary authorities (Service de la sécurité alimentaire et des affaires vétérinaires du canton de Fribourg, authorization numbers: 2016_09_FR and 2019_06_FR).

Note that full information on the approval of the study protocol must also be provided in the manuscript.

Plants

Seed stocks	Not applicable.
Novel plant genotypes	Not applicable.
Authentication	Not applicable.

Magnetic resonance imaging

Experimental design

Design type	Monkeys were positioned prone with arms at their side.
Design specifications	Structural MRI.
Behavioral performance measures	Not applicable.

Acquisition

Imaging type(s): Structural MRI

Field strength: 3T

Sequence & imaging parameters: The MRI parameters of the acquisition were: (1) acquisition matrix: 256x256; (2) 0.7 mm voxel size; (3) echo time (TE) = 3.3s; (4) repetition time (TR) = 7.7s; (5) 3D-T1 and (6) 3D sagittal.

Area of acquisition: Head.

Diffusion MRI: ☐ Used ☒ Not used

Preprocessing

Preprocessing software: Not applicable.

Normalization: Not applicable.

Normalization template: Not applicable.

Noise and artifact removal: Not applicable.

Volume censoring: Not applicable.

Statistical modeling & inference

Model type and settings: Not applicable.

Effect(s) tested: Not applicable.

Specify type of analysis: ☐ Whole brain ☐ ROI-based ☐ Both

Statistic type for inference (See Eklund et al. 2016): Not applicable.

Correction: Not applicable.

Models & analysis

n/a	Involved in the study
☒	☐ Functional and/or effective connectivity
☒	☐ Graph analysis
☒	☐ Multivariate modeling or predictive analysis
